# Gemcitabine, Ifosfamide and Navelbine (GIN): activity and safety of a non-platinum-based triplet in advanced non-small-cell lung cancer (NSCLC)

**DOI:** 10.1054/bjoc.2001.2108

**Published:** 2001-11

**Authors:** E Baldini, A Ardizzoni, T Prochilo, M A Cafferata, L Boni, C Tibaldi, C Neumaier, P F Conte, R Rosso, The Italian Lung Cancer Task Force (FONICAP)

**Affiliations:** U.O. Oncologia Medica Ospedale S. Chiara, via Roma n.67, Pisa, Italy; Divisione di Oncologia Medica I and Servizio di Radiologia, Istituto Nazionale per la ricerca sul cancro, Largo Rosanna Benzi n.10, Genova, Italy; BETA and Advanced Biotechnology Center, Largo Rosanna Benzi n.10, Genova, Italy

**Keywords:** metastatic non-small-cell lung cancer, non-platinum-based regimen

## Abstract

To evaluate activity and toxicity of a non platinum-based triplet including Gemcitabine, Ifosfamide and Navelbine (GIN) in advanced NSCLC. Stage IIIB/IV NSCLC patients with WHO PS < 2 and bidimensionally measurable disease entered the study. Gemcitabine 1000 mg/sqm day 1 and 1000–800 mg/sqm day 4, Ifosfamide 3 g/sqm day 1 (with Mesna), Navelbine 25 mg/sqm day 1 and 25–20 mg/sqm day 4 were administered intravenously every 3 weeks. Objective responses (ORs) were evaluated every 2 courses: a maximum of 6 courses were administered in responding patients. According to Simon's optimal two-stage design more than 18 ORs out of 54 patients were required to establish the activity of this regimen. Fifty patients entered the study. Main characteristics of the 48 evaluated patients were: median age 63 years, ECOG performance status 0 = 65%, stage IV disease 79% and non-squamous histology 71%. The total number of courses administered was 200, median per patient 4 (range 1–6). Toxicities were evaluated according to WHO criteria: neutropenia grade 3–4 occurred in 47% of the courses; thrombocytopenia grade 3–4 in 6.6%; anaemia grade 3 in 3.5%. Twelve episodes of febrile neutropenia were reported and three patients required hospital admission. No toxic death was reported. Non-haematological toxicity, including skin rash, alopecia and fatigue, were generally. Twenty-five ORs (1 complete response and 24 partial responses) were obtained for a response rate of 52% (95% CI: 37.4–66.5%). One-year survival was 46.5%. This non-platinum-based outpatient triplet showed promising activity against NSCLC with myelosuppression, in particular neutropenia, being dose-limiting. The GIN regimen may represent a valuable alternative to standard platinum-based doublets and triplets in the treatment of advanced NSCLC and further studies with this platinum-free combination are warranted. © 2001 Cancer Research Campaign  http://www.bjcancer.com


*British Journal of Cancer* (2001) **85**(10), 1452–1455
© 2001 Cancer Research Campaign
doi: 10.1054/ bjoc.2001.2108, available online at http://www.idealibrary.com on IDEAL

### Gemcitabine, Ifosfamide and Navelbine (GIN): activity and safety of a non-platinum-based triplet in advanced non-small-cell lung cancer (NSCLC)

**E Baldini[1], A Ardizzoni[2], T Prochilo[1], MA Cafferata[2], L Boni[4], C Tibaldi[1], C Neumaier[3], PF Conte[1] and R Rosso[2] on behalf of the Italian Lung Cancer Task Force (FONICAP)**

[1]U.O. Oncologia Medica Ospedale S. Chiara, via Roma n.67, Pisa, Italy; [2]Divisione di Oncologia Medica I and [3]Servizio di Radiologia, Istituto Nazionale per la ricerca sul cancro, Largo Rosanna Benzi n.10, Genova, Italy and [4]BETA and Advanced Biotechnology Center, Largo Rosanna Benzi n.10, Genova, Italy

**Summary** To evaluate activity and toxicity of a non platinum-based triplet including Gemcitabine, Ifosfamide and Navelbine (GIN) in advanced NSCLC. Stage IIIB/IV NSCLC patients with WHO PS < 2 and bidimensionally measurable disease entered the study. Gemcitabine 1000 mg/sqm day 1 and 1000–800 mg/sqm day 4, Ifosfamide 3 g/sqm day 1 (with Mesna), Navelbine 25 mg/sqm day 1 and 25–20 mg/sqm day 4 were administered intravenously every 3 weeks. Objective responses (ORs) were evaluated every 2 courses: a maximum of 6 courses were administered in responding patients. According to Simon's optimal two-stage design more than 18 ORs out of 54 patients were required to establish the activity of this regimen. Fifty patients entered the study. Main characteristics of the 48 evaluated patients were: median age 63 years, ECOG performance status 0 = 65%, stage IV disease 79% and non-squamous histology 71%. The total number of courses administered was 200, median per patient 4 (range 1–6). Toxicities were evaluated according to WHO criteria: neutropenia grade 3–4 occurred in 47% of the courses; thrombocytopenia grade 3–4 in 6.6%; anaemia grade 3 in 3.5%. Twelve episodes of febrile neutropenia were reported and three patients required hospital admission. No toxic death was reported. Non-haematological toxicity, including skin rash, alopecia and fatigue, were generally. Twenty-five ORs (1 complete response and 24 partial responses) were obtained for a response rate of 52% (95% CI: 37.4–66.5%). One-year survival was 46.5%. This non-platinum-based outpatient triplet showed promising activity against NSCLC with myelosuppression, in particular neutropenia, being dose-limiting. The GIN regimen may represent a valuable alternative to standard platinum-based doublets and triplets in the treatment of advanced NSCLC and further studies with this platinum-free combination are warranted. © 2001 Cancer Research Campaign http://www.bjcancer.com

**Keywords**: metastatic non-small-cell lung cancer; non-platinum-based regimen

Non-small-cell lung cancer (NSCLC) represents one of the main causes of cancer death in Western countries and more than three quarters of lung cancer patients become candidates for systemic chemotherapy at some time during the course of their illness. Cisplatin remains the cornerstone of chemotherapy and cisplatin-based regimens have become the gold standard for the management of this disease (Stewart and Pignan, 1995). In addition, data from literature would also suggest the superiority of platinum-based triplets over doublets in selected patients with advanced NSCLC (Crinò et al, 1995; Comella et al, 2000). However, cisplatin represents the drug with the highest negative impact in terms of patient compliance and workload for care givers; after four to six cycles of chemotherapy, the cumulative neurotoxicity of cisplatin-containing regimens, often becomes severe and irreversible (Sculier et al, 1994). This could be considered less important in patients with metastatic disease having short-term poor prognosis but, for patients with earlier stage of disease and higher probability of long-term survival, cumulative neurotoxicity is particularly relevant as it can strongly affect the quality of life. In addition, the administration of high-dose cisplatin, is often associated with acute and late gastrointestinal toxicities (severe nausea/vomiting and diarrhoea) and requires hospitalization for hyperhydration and forced diuresis.

Over the last 10 years a large number of new active agents have emerged, including taxanes, gemcitabine, navelbine, which have been incorporated into cisplatin-based regimens. However, despite encouraging response rates, no significant survival advantage could be consistently) demonstrated; furthermore, acute and cumulative toxicities of these new platinum-based doublets and triplets were superimposable, or even worse, than those observed with second generation regimens (Giaccone et al, 1998; Bonomi et al, 2000; Crinò et al, 1999). Nevertheless, the availability of new active agents along with the need for better tolerated combination regimens fostered further investigation in the area of non-platinum chemotherapy regimen.

On these grounds, we performed the present phase II study aimed at defining the antitumour activity and toxicity profile of a non-platinum-based triplet consisting of Gemcitabine, Ifosfamide and Navelbine in advanced NSCLC patients.

Gemcitabine is a nucleoside analogue with a novel mechanism of action that showed reproducible response rate of approximately 20% in patients with untreated NSCLC. At standard doses, the weekly administration of Gemcitabine is associated with a low incidence of side-effects, in particular myelosuppression, nausea, vomiting and alopecia (Sheperd, 1995). Ifosfamide is a well known alkylating agent that binds to DNA causing strand breaks: it showed antitumour activity against NSCLC with response rates

*Received 1 June 2001*
*Accepted 30 July 2001*

*Correspondence to:*

ranging from 20 to 32% when administered as single agent in previously untreated patients (Eberhardt and Niederle, 1992). Navelbine is a semisynthetic vinca alkaloid that demonstrated less neurologic toxicity than the parental compounds mainly due to its selectivity for mitotic cells over axonal microtubes. Its antitumor activity and efficacy have been very well demonstrated in phase II and III clinical trials as single agent or in combination with platinum compounds (Crawford, 1996). The significant antitumor activity along with the favourable toxicity profile of these agents prompted their combined use in a platinum-free regimen

### PATIENTS AND METHODS

### Eligibility

Chemotherapy-naive patients with histologically or cytologically proven NSCLC and an Eastern Cooperative Oncology Group (ECOG) performance status (PS) < 2 were included. Eligible patients were required to have bidimensionally measurable stage IIIB disease (pleural effusion and/or supraclavicular nodes) or metastatic disease. Adequate pre-treatment haematologic (WBC count ≤ 4000/µl, hemoglobin ≥ 10 g/dl), hepatic (bilirubin < twice above normal level) and renal (creatinine < twice above normal limit) functions were also mandatory. Patients with symptomatic brain metastases were excluded. Staging procedures consisted of: bronchoscopy, chest X-ray, CT scan, abdominal ultrasound performed within 4 weeks before the beginning of the treatment; other types of organ-specific scanning were optional but recommended in cases of symptoms or biochemical abnormalities. Restaging was planned every two courses of chemotherapy: all target lesions were reassessed with the same method used at study entry according to WHO criteria (Miller et al, 1981). All responses were extramurally reviewed by an expert radiologist who was not aware of the type of treatment; objective remissions had to be confirmed 4 weeks apart. Patients were evaluated weekly for toxicity according to WHO recommendations during the whole therapeutic program.

Written informed consent was obtained from all patients according to local institution policies: the study was approved by the local IRB/ethical committee of participating institutions and was conducted according to GCP. The study was sponsored and monitored by Eli-Lilly Italy, while registration, data entry, management and analysis were carried out independently at the National Cancer Institute for Cancer Research in Genoa.

The original chemotherapy protocol consisted of: navelbine 25 mg/sqm (as slow intravenous bolus) day 1 and day 4, gemcitabine 1000 mg/sqm days 1 and 4 and ifosfamide 3 g/sqm (infused over 2 h) day 1. Mesna (sodium 2-mercaptoethane sulfonate) was administered as uroprotection at the following doses: 20% of the total ifosfamide dose intravenously, immediately before the drug infusion, and 40% of the total dose orally after 4 and 8 h. Since three cases of neutropenic fever were recorded among the first 8 cases enrolled, the protocol was amended by reducing the dose of navelbine and gemcitabine on day 4 to 20 mg/sqm and 800 mg/sqm respectively. Courses were repeated every 3 weeks on an outpatient basis. All patients received intravenous anti-HT3 antagonists and dexamethasone as anti-emetic prophylaxis. White blood cell counts (WBC) were performed weekly and biochemistry was determined on day 1 of each cycle; chemotherapy on day 4 was administered with no WBC determination. In the case of grade 4 neutropenia, ciprofloxacin 500 mg orally twice a day and fluconazole 100 mg daily were administered until ANC > 1000/µl. The prophylactic use of colony stimulating factors (CSFs) was not allowed while their therapeutic use was suggested for febrile neutropenia. In case of grade 3/4 thrombocytopenia or febrile neutropenia a 25% reduction in the doses of the three drugs administered on day 1 was performed in subsequent courses of chemotherapy.

Simon's optimal two-stage design for phase II clinical trials was used to calculate the sample size (Simon, 1989): P0 (clinically uninteresting true response rate) and P1 (sufficiently promising true response rate) were set at 20% and 40% respectively. In the first stage 19 patients had to be included: if ≤ 4 responses were observed, accrual was stopped, otherwise 35 more patients had to be registered. Drug combination was considered of interest if > 18 responses were observed out of 54 evaluable patients. Survival was measured from the date of registration to death. Patients still alive at the time of the final analysis were censored at the date in which they were last observed. Progression-free survival was calculated from the date of registration to the date of clinical and/or radiological evidence of progression or death, whichever occur first. Patients not progressed at the time of the final analysis were censored at the date of their last tumour assessment. Survival and time to progression were estimated using the Kaplan-Meier method (Kaplan and Meier, 1985).

### RESULTS

From March 1999 to March 2000, 50 chemotherapy-naive patients from three participating institutions were registered. Accrual into this study was stopped as soon as the number of responses required by the statistical design was achieved. Two patients were excluded from the analysis (one was inelegible because of age > 70 and the other was never treated). Characteristics of the remaining 48 evaluated patients were as follows: median age 63 years (range 44–69); ECOG performance status 0 = 64.6%, 1 = 35.4%; the majority of patients had stage IV disease (79%) and non-squamous histology (70.8%). Patient characteristics are listed in Table 1.

### Toxicity

Toxicity data are reported in Table 2 as the worst toxicity grade experienced per course at any time in the trial. A total of 200 courses of chemotherapy were administered median per patient 4 (range 1–6). Myelosuppression was the most frequent side-effect: grade 4 neutropenia was observed in 21.6% of the courses, while grade 4 thrombocytopenia affected 2.5% of chemotherapy courses

**Table 1** Patient characteristics

| Characteristic | *n* | (%) |
|---|---|---|
| Total | 48 | |
| Median age years (range) | 63 (44–69) | |
| ECOG PS | | |
| 0 | 31 | 64.6 |
| 1 | 17 | 35.4 |
| Stage | | |
| IIIB | 10 | 20.8 |
| IV | 38 | 79.2 |
| Histology | | |
| Squamous | 14 | 29.2 |
| Non-squamous | 34 | 70.8 |

© 2001 Cancer Research Campaign

**Table 2** Worst toxicity per course (WHO)

| | G3% | G4% |
|---|---|---|
| Leucopenia | 20.5 | 6.6 |
| Neutropenia | 25.2 | 21.6 |
| Thrombocytopenia | 4.1 | 2.5 |
| Anaemia | 3.5 | / |
| Vomiting | 2.5 | / |
| Skin rash | 1.3 | / |
| Alopecia | 4.6 | / |

only. Twelve episodes of febrile neutropenia were observed, including three cases among the first 8 patients treated at the highest protocol dose; three patients were admitted for parenteral antibiotic treatment and one of these discontinued the treatment after the 4th course of chemotherapy. Three additional patients interrupted the therapeutic programme early: two because of worsening performance status after the 4th and 5th course respectively and one patient because of severe skin rush requiring corticosteroid treatment after the second course. However, compliance to the treatment was good in the majority of patients and the average delivered over planned chemotherapy dose was 98%. No toxic deaths were observed.

### Response and survival

In the first step, 10 out of 19 patients obtained a major objective remission; according to the statistical design we proceeded to the second step where 25 objective responses (24 partial responses and 1 complete response) were obtained from the first 48 evaluated patients with a response rate of 52% (95% C.I.: 37.4–66.5%). Accrual was stopped at this point as we had already overcome the figures we needed. Fourteen patients had stable disease (29%) while 6 progressed during chemotherapy. Three patients were not evaluable for response as they had received less than two courses of chemotherapy: however, according to the 'intention to treat' policy, they were included in the response analysis as failures. All objective remissions were extramurally reviewed and confirmed at least 4 weeks apart. The median time from treatment onset and response documentation was 6 weeks (range 6–12). The median time to progression was 7.1 months (95% CI: 5.5–9.9) and median overall survival was 11 months (95% CI: 9.6-NA) (Figure 1). One-year survival was 46.5%.

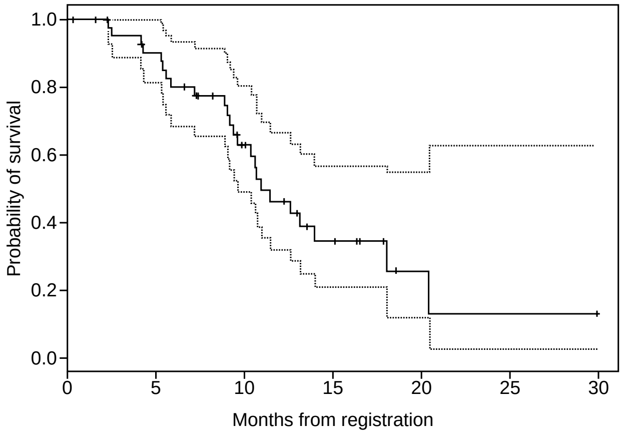

**Figure 1** Overall survival curve

### DISCUSSION

The current availability of new active drugs for the treatment of NSCLC provided an impulse for the investigation of cisplatin-free regimens with high antitumour activity and good tolerability. In the present multi-centre phase II study, three drugs with single-agent activity against NSCLC and favourable toxicity profile, gemcitabine, ifosfamide and navelbine, were combined, for the first time, in a phase II study in patients with advanced disease. The choice of the drug-combination tested in this study was based on encouraging results obtained in a previous experience of our group with the triplet Vinorelbine, Ifosfamide, Cisplatin (VIP) in metastatic and locally advanced inoperable disease (Baldini et al, 1996; Baldini et al, 2000). The replacement of Cisplatin with Gemcitabine, was carried out to reduce cisplatin-related acute and long-term cumulative side-effects and to avoid hyperhydration and enforced diuresis, while maintaining high antitumour activity.

The schedule used in this phase II study, with navelbine and gemcitabine on days 1 and 4, was chosen in order to maintain the dose-intensity of each drug: in fact, the weekly schedule of navelbine, because of the overlap between drug dosing and WBC nadir, often requires dose reductions (Gralla et al, 1998). The schedule day 1 to day 4 allowed the delivery of full doses of the two drugs, with no further reduction due to haematologic toxicity: the average actual dose of chemotherapy was 98% of the planned dose, with good treatment compliance for the majority of patients.

As expected, acute haematologic toxicity was the predominant side-effect of this regimen. However, grade 3–4 neutropenia was generally short-lasting and febrile neutropenia, occurring in 6% of the courses, was successfully managed on an outpatient basis in most cases. None of the patients experienced peripheral neuropathy or renal failure, while 9 out of 48 (18%) patients experienced cumulative toxicity in the form of grade 3 fatigue.

The excellent antitumour activity of this novel triplet emerges from the early interruption of accrual once the number of responses required by the statistical design of the study was overcome. To our knowledge, this is the first phase II trial with the GIN regimen in NSCLC. Our results compare favourably in terms of activity and tolerability with those of novel cisplatin-based doublets (Crinò et al, 1997; Pirker et al, 1995) and triplets (Baldini et al, 1996; Comella et al, 1999). In addition, our figures appear superior to those of doublets including the same agents as in our regimen (Gridelli et al, 2000; Morere et al, 1997). Activity of the GIN regimen also compares favourably with that of other non platinum-based triplets (Gralla et al, 1998). The activity of the GIN regimen is particularly interesting considering that, in our series, the majority of patients had metastatic non squamous lung tumours suggesting that cisplatin may not be an essential component in the chemotherapy of these histological subtypes. In vitro experiences demonstrated that drugs such as topotecan and cisplatin are particularly active in squamous cell carcinoma cell lines while other drugs, including taxanes and gemcitabine, appear to have a higher antitumour activity in non-squamous tumors (Loprevite et al, 1999). These in vitro data are in keeping with some clinical observations suggesting a relationship between histologic subtype and the probability of response to platinum-free or -based regimens (Georgoulias et al, 2000; Boni et al, 1997). The possibility of considering different chemotherapy regimens (platinum vs non-platinum based) for different histological subtype in the treatment of non-small cell lung cancer should be further explored.

In conclusion, a non-cisplatin based chemotherapy regimen based on a combination of gemcitabine, ifosfamide and navelbine, has

 *© 2001 Cancer Research Campaign*

high activity and acceptable toxicity in the treatment of advanced NSCLC and deserves further investigation within prospective randomized studies assessing the role triplets, with or without platinum, in the treatment of advanced non-small-cell lung cancer.

### REFERENCES

Baldini E, Tibaldi C, Chella A, Angeletti CA, Silvano G, Andrei A, Algeria R and Conte PF (1996) Phase II study of vinorelbine/ifosfamide/cisplatin for the treatment of advanced non-small-cell lung cancer. *Ann Oncol* **7**: 747–749

Baldini E, Silvano G, Tibaldi C,Campoccia S, Cionini L and Conte PF (2000) Sequential chemoradiation therapy with vinorelbine, ifosfamide and cisplatin in stage IIIB non-small-cell lung cancer: A phase II study. *Semin Oncol* **27** (**Suppl 1**): 28–32

Boni L, Pennucci MC, Ardizzoni A et al (1997) Factors affecting response rate (RR) and survival (OS) in advanced non-small-cell lung cancer (NSCLC): retrospective analysis on 1128 patients. *Lung Cancer* **18** (**Suppl 1**): 5

Bonomi P, Kyungmann K, Fairclough D, Cella D, Kugler J, Rowinsky E, Jiroutek M and Johnson D (2000) Comparison of survival and quality of life in advanced NSCLC patients treated with two dose-levels of paclitaxel combined with cisplatin vs etoposide with cisplatin: an Eastern Cooperative Oncology Group (ECOG) trial. *J Clin Oncol* **18**: 623–631

Comella P, Frasci G, Panza N, Manzione L, Lorusso V, Di Rienzo G, Cioffi R, De Cataldio G, Maiorino L, Bilancia D, Natale M, Carpagnano F, Pacilio C, De Lena M, Bianco A and Comella G (1999) Cisplatin, gemcitabine and vinorelbine combination therapy in advanced non-small-cell lung cancer: a phase II randomized study of the Southern Italy Cooperative Oncology Group. *J Clin Oncol* **17**: 1526–1534

Comella P, Frasci G, Panza N, Manzione L, De Cataldo G, Cioffi R, Maiorino L, Micillo E, Lorusso V, DiRienzo G, Filippelli G, Lamberti A, Natale M, Bilancia D, Nicolella G, Di Nota A and Comella G (2000) Randomized trial comparing cisplatin, gemcitabine and vinorelbine with either cisplatin and gemcitabine or cisplatin and vinorelbine in advanced non-small-cell lung cancer: interim analysis of a phase II trial of the Southern Italy Cooperative Group. *J Clin Oncol* **18**: 1451–1457

Crawford J (1996) Vinorelbine in the treatment of non-small-cell lung cancer. *Semin Oncol* **23** (**Suppl 5**): 2–8

Crinò L, Clerici M, Figoli F, Carlini P, Ceci G, Cortesi E, Carpi A, Santini A, Di Costanzo F, Boni C, Meacci M, Corgna E, Darwish S, Scarcella L, Santucci A, Ballatori E and Tonato M (1995) Chemotherapy of advanced non-small-cell lung cancer: a comparison of three active regimens: A randomised trial of the Italian Oncology Group for Clinical trial research (GOIRC). *Ann Oncol* **6**: 347–353

Crinò L, Scagliotti G, Marangolo M, De Marinis F, Rinaldi M, Gridelli C, Ciribelli A, Bianco R, Marangolo M, Di costanzo F, Sassi M, Barni S, Ravaioli A, Adamo V, Portalone C, Cruciani G, Masotti A, Ferrarar G, Gozzellino f and Tonato M (1997) Cisplatin-gemcitabine combination in advanced non-small-cell lung cancer: a phase II study. *J Clin Oncol* **15**: 297–303

Crinò L, Scagliotti GV, Ricci S et al (1999) Gemcitabine and cisplatin versus mitomycin, ifosfamide, and cisplatin in advanced non-small-cell lung cancer: a randomized phase III study of the Italian Lung Cancer Project. *J Clin Oncol* **17**: 3522–3530

Eberhardt W, Niederle N (1992) Ifosfamide in non-small cell lung cancer: a review. *Semin Oncol* **19** (**Suppl 1**): 40–48

Frasci G, Panza N, Manzione L (2000) Randomized trial comparing cisplatin, gemcitabine and vinorelbine with either cisplatin and gemcitabine or cisplatin and vinorelbine in advanced non-small-cell lung cancer: interim analysis of a phase III trial of the Southern Italy Cooperative Group. *J Clin Oncol* **18**: 1451–1457

Georgoulias V, Papadakis E, Alexopulos A, Tsiafakis X, Rapti A, Veslemes M, Palamidas P, Vlachonikos I (2000) Docetaxel plus cisplatin versus docetaxel plus gemcitabine in advanced non-small-cell lung cancer: a preliminary analysis of a multicenter randomized phase II trial. *Proc 10th International Congress on Anticancer Treatment* (abstr 14), 82–83

Giaccone G, Splinter TA, Debruyne C, Kho GS, Lianes P, van Zandwijk N, Pennucci MC, Scagliotti G, van Meerbeeck J, van Hoesel Q, Curran D, Sahmond T and Postmus PE (1998) Randomized study of paclitaxel-cisplatin versus cisplatin-teniposide in patients with advanced non-small-cell lung cancer. The European organization for Research and Treatment of Lung Cancer Cooperative Group. *J Clin Oncol* **16**: 2133–2141

Gralla RJ, Rittenberg CN, Silverman CB,Maeques CB, Maulick JF, Cole JT (1998) Gemcitabine + vinorelbine in combination with mitomycin in non-small-cell lung cancer (NSCLC): results of a phase I and phase II study of an active outpatient regimen. *Proc Am Soc Clin Oncol* **17**: 486 (abstr 1871)

Gridelli C, Frontini L, Perrone F, Gallo C, Gulisano M, Cigolari S, Castiglione F, Robbiati SF, Gasparini G, Iannello GP, Farris A, Locatelli MC, Felletti R and Piazza E (2000) Gemcitabine plus vinorelbine in advanced non-small-cell lung cancer: a phase II study of three different doses. *Br J Cancer* **83**: 707–714

Kaplan EL, Meier P (1998) Non parametric estimation from incomplete observation. *J Am Stat Assoc* **53**: 457–481

Loprevite M, Favoni RE, De Cupis A, Pirani P, Merlo F, Grosssi F and Ardizzoni A (1999) Pre-clinical evaluation of new antineoplastic agents in NSCLC cell lines: evidence of histological subtype-dependent cytotoxicity. *Int J Oncol* **15**: 787–792

Miller AB, Hoogstraaten B, Staquest M (1981) Reporting results of cancer treatment. *Cancer* **47**: 207–214

Morere F, Piperno S, Brunet A, Boaziz C, Khon M, Bouillet T and Breau JL (1997) Vinorelbine and ifosfamide for unresectable non-small-cell lung cancer. *Lung Cancer* **18**: 95–100

Pirker R, Krajnik G, Zochbauer S, Malayeri R, Kneusssl M, Huber H (1995) Paclitaxel/cisplatin in advanced non-small-cell lung cancer (NSCLC). *Ann Oncol* **6**: 833–835

Sculier JP, Klastersky J, Giner V, Bureau G, Thiriaux J, Dabouis G, Efremidis A, Ries F, Berchier MC and Sergysels R (1994) Phase II randomized trial comparing high-dose cisplatin with moderate-dose cisplatin and carboplatin in patients with advanced non-small cell lung cancer. *J Clin Oncol* **12**: 353–359

Sheperd FA (1995) Phase II trials of single-agent activity of gemcitabine in patients with advanced non-small-cell lung cancer: an overview. *Anticancer Drug* **6**: 19–25

Simon R (1989): Optimal two-stage design for phase II clinical trials. *Controlled Clin Trials* **10**: 1–10

Stewart LA and Pignon JP (1995) Chemotherapy in non-small cell lung cancer. A meta-analysis using updated individual patients data from 52 randomized clinical trials. *BMJ* **311**: 899–909

© 2001 Cancer Research Campaign
